# Dino Diets Revealed by Isotopes

**DOI:** 10.1021/acscentsci.6c00222

**Published:** 2026-02-18

**Authors:** Rachel Brazil

## Abstract

What did dinosaurs such as ‘Spinosaurus’ eat? Analytical techniques settle some decades-long debates.

## Graphical abstract

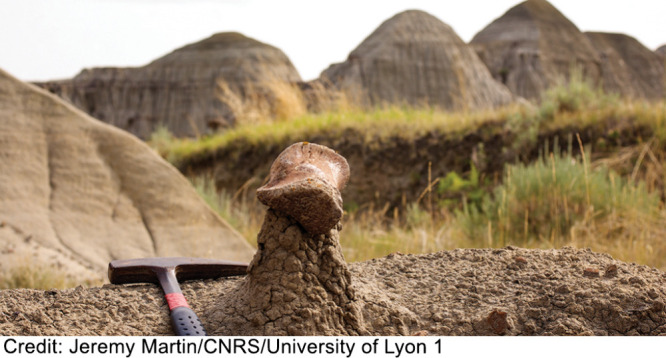

In *Jurassic Park III*, humans are pitted against the massive *Spinosaurus*. The creature is 13 m long and has a crocodile-like tail and a large back sail; the movie shows it running on two legs and fighting the land-based *Tyrannosaurus rex*.

Jeremy Martin, a paleobiologist at the French National Center for Scientific Research (CNRS) and the University of Lyon 1, says the depiction is nonsense: Spinosaurus was not a land predator at all but predominantly ate fish.

This insight about *Spinosaurus*’s diet is based partly on Martin’s work. By analyzing isotope ratios in dinosaur fossils, he can get a picture of what dinosaurs ate.

Ecologists have long used nonradiogenic carbon and nitrogen isotopes to reconstruct the diets of animals in today’s ecosystems. But neither of those elements has historically proved useful for dinosaurs: the carbon isotope ratio isn’t relevant in their ancient diet, and the organic material containing nitrogen isotopes typically doesn’t survive long enough to be analyzed. The dino breakthrough didn’t come until the 1990s, when paleoanthropologists began analyzing calcium isotopes in fossils to learn more about the hominin diet. Paleontologists realized they could also use calcium to probe the diet of dinosaurs at least 66 million years back.

**Figure:**
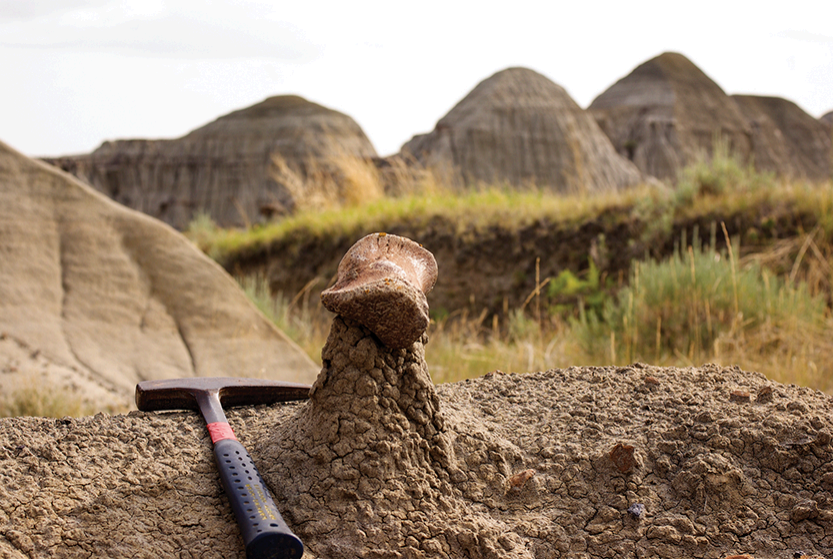
A dinosaur bone sits on top of a spire of rock exposed by erosion in the Dinosaur Park Formation in Alberta. Researchers analyze the ratios of calcium isotopes in bones and fossils to learn more about what dinosaurs once ate. Credit: Jeremy Martin/CNRS/University of Lyon 1.

“When I published some of my first papers on this, I would frequently get reviewers saying this is all mumbo jumbo, none of this is possibly preserved,” says Thomas M. Cullen, a paleobiologist at Auburn University. His results over the last 15 years have shown otherwise.

Today, a community of geochemists and paleontologists are analyzing isotope ratios of calcium along with a broader range of elements to learn more about what dinosaurs ate when they roamed the earth 66 million–252 million years ago. The scientists are sampling dinosaur remains from the many sites still abundant with fossils, such as the Dinosaur Park Formation, a Late Cretaceous floodplain in Alberta.

And thanks to a creative new method, some scientists are revisiting the possibility of tapping into nitrogen isotopes. Their discoveries are helping build a better picture of dinosaur ecosystems and tackle some unanswered questions, including why dinosaurs became extinct.

## Which isotopes remain?

In modern ecosystems and going back 30 million years, the ratio of ^13^C to ^12^C provides an indicator of the source of plant matter consumed at the bottom of a food chain. The difference in ratios is due to plants taking up carbon dioxide for photosynthesis in different ways. Plants like millet or maize (C4 plants) end up with a higher proportion of ^13^C than plants such as rice, wheat, and potato (C3 plants).

For dinosaurs, this ratio does not provide as much insight. More than 30 million years ago, “C4 plants are not really around” in the environments that dinosaurs lived in, “so everything has that same C3 baseline,” Cullen says.

**Figure:**
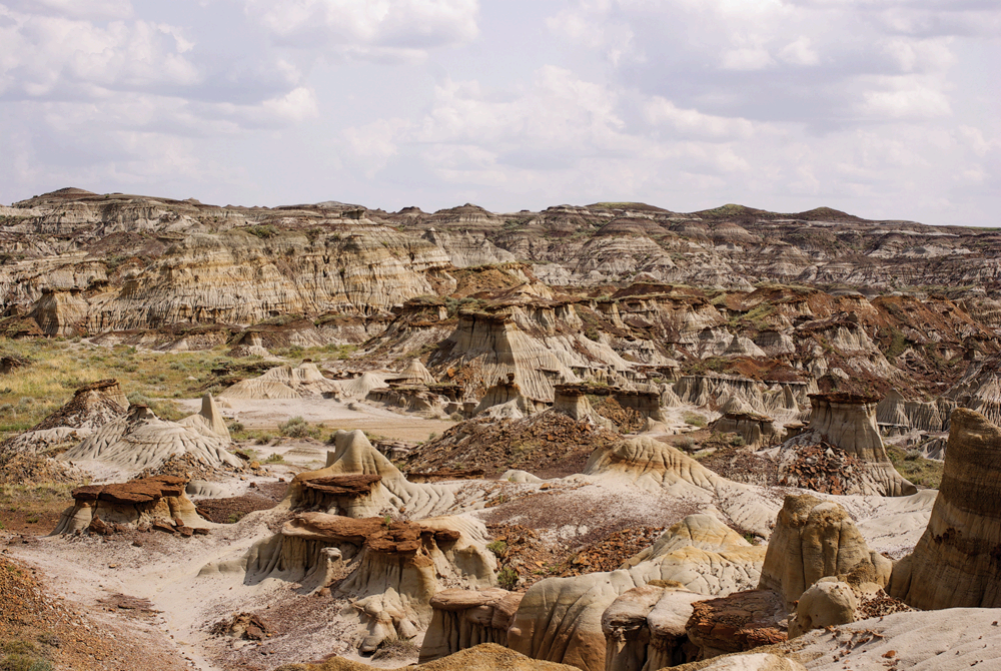
Dinosaur Park Formation. Stacks of alternating sandstone and mud dating back 75 million years are rich pickings for dinosaur bones and teeth. Credit: Jeremy Martin/CNRS/University of Lyon 1.

The other crucial element in reconstructing diets is nitrogen. Scientists use it to determine an animal’s trophic level—whether it is a herbivore, carnivore, or apex predator. While there can be small differences in how nitrogen isotopes are taken up from the soil in plants, the more-significant differences are at the higher trophic levels. This is because amino acids containing the lighter nitrogen isotope, ^14^N, are more quickly broken down and excreted by animals, which results in a greater ratio of ^15^N to ^14^N. At each subsequent step in the food chain—for instance, going from a herbivore to a carnivore—the ratio of ^15^N to ^14^N increases.

Unfortunately, organic material is unlikely to survive in anything older than 100,000 years, so the nitrogen isotope ratio is another thing that hasn’t historically helped in analyzing dinosaur diets. What does survive much longer is hydroxyapatite, the calcium phosphate nanocrystalline material that makes up 70% of bone. In hydroxyapatite, researchers can trace the ratio of ^44^Ca to ^42^Ca.

Like the carbon and nitrogen ratios, this calcium ratio provides a diet proxy. In this case, the lighter calcium isotopes are preferentially incorporated, taken up by protein-mediated transport through the gut walls; the heavier isotopes are preferentially excreted in the kidneys. So carnivorous dinosaurs, whose calcium would have come from eating animals already enriched in the lighter calcium isotope, would have lower ^44^Ca to ^42^Ca ratios than their herbivorous prey.

An issue with using calcium isotopes is that the ratios could change over time. In a process known as diagenesis, natural fluids that enter burial sediments start to dissolve the hydroxyapatite nanocrystals, which then recrystallize. “If there is material exchange with the ambient soil solution, then obviously you change the original biogenically incorporated trace element or isotope signatures,” says Thomas Tütken, a geochemist and paleontologist at Johannes Gutenberg University Mainz.

To get around this problem, the vast majority of current studies rely on dinosaur tooth enamel, which is also hydroxyapatite. “The crystallites are an order of magnitude larger than in bones,” Tütken says. They therefore have less surface area and will better resist dissolution by stray fluids, and the tooth’s calcium isotopes will stay truer to the original.

## Analyzing ecosystems

By unearthing data about dinosaur diets from the calcium isotope ratios, researchers are piecing together fundamental information on dinosaur ecosystems. For example, they now have a better idea of how some different dinosaurs coexisted—for instance, whether they were competing for food or had their own niche.

A recent study of calcium isotope ratios from dinosaur tooth enamel from the Late Jurassic Period (161.5 million–145 million years ago) found at Dinosaur National Monument on the border between Colorado and Utah gives clues to how different dinosaur species lived together there. Researchers found that sturdy, long-necked *Camarasaurus* had statistically distinct calcium isotope ratios from the beaked, four-fingered *Camptosaurus*. This shows that their diets were different, although they lived in the same environment: *Camarasaurus* preferred woody plant tissue and conifers, while *Camptosaurus* ate softer leaves and buds.

Calcium isotope ratios are also helping solve some long-running debates that the fossil record can’t explain, such as the feeding pattern of *Spinosaurus*. “There is a hot debate about his position in the environment—if it’s aquatic or if it’s a land animal,” Martin says. This huge dinosaur lived in North Africa about 100 million years ago. In 2018, Martin and colleagues looked at calcium isotope ratios from tooth enamel of *Spinosaurus* and other co-occurring large predators excavated from sites in Niger and Morocco.

**Figure:**
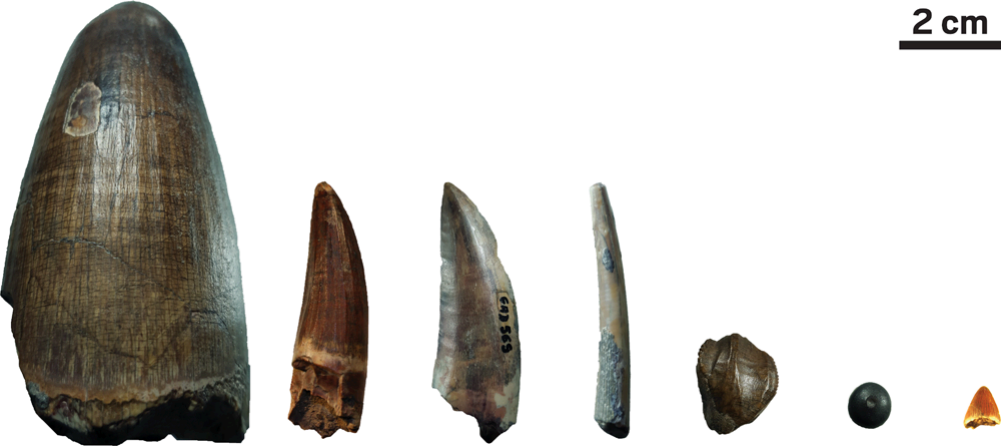
A collection of dinosaur teeth from Niger’s Gadoufaoua deposit dating from 120 million years ago and used by Jeremy Martin and colleagues to measure calcium isotope ratios. From left to right: Teeth of a giant crocodile, *Sarcosuchus imperator*; a spinosaurid (carnivore); a nonspinosaurid theropod (abelisaurid or carcharodontosaurid, both carnivores); a pterosaur (herbivore); a hadrosaurid (herbivore); a pycnodont (fish); and a small crocodylomorph (carnivore). The scale bar represents 2 cm. Credit: Auguste Hassler/University of Aberdeen/University of Ottawa.

“The results we obtained showed that the *Spinosaurus* were very depleted in heavy calcium, and it was something a bit unexpected, because why would they be so different from the rest?” Martin says. The team’s best explanation is that *Spinosaurus* were eating fish, which is lower in heavy calcium than land-based animals. Martin says this is the sort of niche partitioning—similar species coexisting in an environment using very different resources—that the group aims to learn more about. Before isotope ratio analysis, researchers understood little of how the animals shared resources within the ecosystem.

## Other isotope systems

The calcium isotope ratio is not a flawless metric for understanding diets. One difficulty is that the differences in the calcium isotope ratio between trophic levels are smaller than the differences in the nitrogen isotope ratio, which makes them sometimes confusing to interpret.

Because of this challenge, some scientists are looking at other isotope ratios to get a wider picture of the diets of some ancient animals. The most helpful isotopes so far are zinc. “I was very skeptical about it, because it’s a trace metal. It’s also known to change in diagenic processes,” Tütken says. But he has been surprised to find that the zinc isotope ratio is still useful, as zinc is incorporated into the outer layers of tooth enamel during its formation, thus substituting for the calcium in hydroxyapatite.

The ^66^Zn to ^64^Zn ratio in the food chain is more complicated than the ratio of some other elements. “The heavier isotope is preferentially bound to ligands with a stronger electronegativity, like oxygen, and then you get more of the lighter isotopes when it binds to ligands with nitrogen or sulfur,” says Jeremy McCormack at Goethe University. Zinc in plants is bound primarily via organic acids, meaning the heavier isotope is dominant. In animals, zinc often binds to the sulfur in amino acids, which results in more of the lighter isotope as you go up a food chain.

McCormack and Tütken found the zinc isotopes particularly useful when they collaborated with others on a study of feeding patterns in huge sharks, including some that existed 18 million years ago. Shark teeth are made of a fluorapatite mineral known as enameloid and are high in zinc.

In that work, the researchers compared extinct megalodon sharks and other ancient species to a selection of younger species like the great white, whose trophic position and diet are known. They found variability in the megalodon diet “at exactly the same trophic position,” McCormack says. “They were opportunistic predators feeding on whatever was available to the population at the time—which of course makes sense, because that’s exactly what we see in modern sharks as well.” The similarities in the shark species’ feeding habits would put them in direct competition and could also explain why the megalodon became extinct. Tütken is optimistic that he and McCormack will next be able to analyze zinc isotopes ratios for dinosaur teeth.

Tütken has also used the ratio of ^88^Sr to ^86^Sr in hydroxyapatite to study many large herbivore species native to North America in the Cretaceous Period (100 million–66 million years ago) and look for signs of competition versus coexistence. Isotope data from samples found in a site in the Upper Cretaceous Oldman Formation showed that duck-billed hadrosaurs had different diets than other herbivores, such as armored ankylosaurs and the horned ceratopsians. Tütken suggests that the different isotopic signatures might be explained by the dinosaurs feeding at different heights and on different parts of a plant.

## A new method for nitrogen isotope analysis

Despite the new isotopes that researchers are trying to leverage, it would be beneficial if they could access even a small stash of nitrogen compounds that dinosaur fossils keep intact. A new method developed by Jennifer Leichliter and Tina Lüdecke at the Max Planck Institute for Chemistry could allow scientists to do that.

Nitrogen is useful because the shifts in the ratio between trophic levels are larger, making it much easier for scientists to distinguish differences between a herbivore, a carnivore, and a top predator. For nitrogen, the ratio shifts 3–5 parts per thousand as you move between trophic levels; for calcium, the shift is only 0.63 parts per thousand.

In the past 5 years, Leichliter and Lüdecke have worked on a method that extracts new information from dinosaur teeth—specifically, from the less than 1% of 1% that is composed of nitrogen. Unlike the nitrogen from other parts of a dinosaur’s body, including bone, the nitrogen in tooth enamel seems to survive over millions of years. “It’s the compact crystalline structure of tooth enamel and the very high degree of mineralization that is, we think, preserving in most cases that endogenous organic matter,” Leichliter says.

There is not enough nitrogen present in the tooth enamel to be detected using the conventional mass spectrometry approach; because there is so little of it, the atmospheric nitrogen background interferes. Instead, Leichliter and her collaborators have repurposed a method originally designed to measure nitrogen levels in small samples of ocean water. They first oxidize the sample to convert all nitrogen into nitrates. They then use bacteria to convert the nitrates into nitrous oxide gas, which they feed directly into a mass spectrometer. This oxidation-denitrifier method can pick up differences as small as 1–2 nmol of nitrogen for each isotope.

Leichliter first used the technique to analyze 3.5-million-year-old hominin tooth samples but wondered if she could go even further back in time. In collaboration with Tütken, she is now testing the method on dinosaur teeth. One question they are keen to examine and that has proved tricky with other isotope systems is whether carnivorous dinosaurs ate each other or even practiced cannibalism on their own species. “You would see an additional trophic level above the carnivores” if top carnivores were eating other top carnivores, which would probably indicate the species preyed on itself, Leichliter says.

Leichliter, Lüdecke, and Tütken have presented some preliminary results based on fossils from three North American sites up to 150 million years old, including the Dinosaur Park Formation. “We have beautiful trophic preservation—carnivores are higher than herbivores,” Leichliter says, though so far they have found no signal that would indicate cannibalism. They have shown that the method works for reconstructing trophic structure in past eras. “This is just opening the door, peeking through the curtains in terms of what we can then do,” Leichliter adds.

**Figure:**
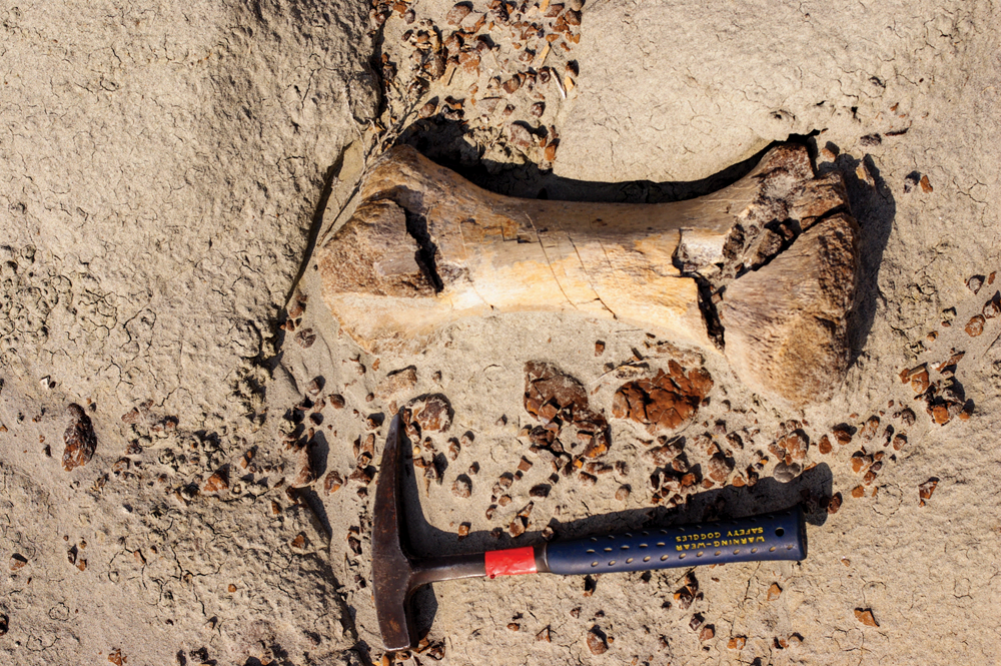
Dinosaur limb bone discovered in soft sediment at the Dinosaur Park Formation. Credit: Jeremy Martin/CNRS/University of Lyon 1.

There are still challenges with nitrogen isotope data alone. Because of the varying baselines of nitrogen isotope ratios in different soils and plants, scientists could misinterpret the data when trying to extrapolate diets.

One solution that researchers at the Max Planck Institute are currently developing is measuring only the isotopic ratio of trophic amino acids, the subset of protein-building amino acids like glutamic acid, which are broken apart and resynthesized during metabolism. Analyzing the amino acids that have the biggest shifts in their isotopic ratios can provide a clearer view than looking at the average across all the amino acids.

“It’s an approach that does exist in modern samples, and it would be cool if it could be applied to fossil samples,” Leichliter says. But if that’s possible remains to be seen.

For now, Cullen and others are excited at the questions they want to tackle with isotopes. For example, did each species have the same diet throughout their lives? “A tyrannosaur starts out from an egg when they’re born, and they end up the size of a large school bus, so they go through a pretty wide range of size change,” he says.

It’s probable that young dinosaurs would start feeding on insects or smaller lizards rather than taking down large herbivores. If researchers can spot age-related diet differences, it will also tell them whether adult dinosaurs were feeding their young or left them to fend for themselves—a fundamental and still-unanswered question.

Researchers might even be able to use isotope ratios to help explain the dinosaur extinction that occurred around 66 million years ago. While it was most likely linked to an asteroid impact, CNRS’s Martin says some researchers think the dinosaurs were already on the decline. Looking at calcium isotopes for changes in dinosaur food chains directly before the extinction could support or refute this idea. Preliminary work hasn’t shown any changes in the food chain before the asteroid, but Martin says it’s still a question he wants to probe.

Scientists do not examine isotopic data in isolation; it is one resource they use to understand Mesozoic Era ecosystems, of which diet is an important component. Paleontologists continue to debate whether *Spinosaurus* lived on land or in water. Martin doesn’t think it was fully aquatic. He says that from isotopic ratios, “the only thing I can tell is that its food source needed to be fish.” And in reality, *Spinosaurus* and *Tyrannosaurus* lived on different continents—the former in Africa and the latter in North America, 30 million years later. But perhaps *Jurassic Park* is entitled to a bit of artistic license.

*Rachel Brazil is a freelance contributor to* Chemical & Engineering News, *the independent news outlet of the American Chemical Society.*

